# Apigenin and Ampicillin as Combined Strategy to Treat Severe *Streptococcus suis* Infection

**DOI:** 10.3390/molecules26071980

**Published:** 2021-04-01

**Authors:** Hao Lu, Chenchen Wang, Wenjia Lu, Xiaodan Li, Zhaoyuan Wu, Gaoyan Wang, Wenqi Dong, Chen Tan, Manli Liu

**Affiliations:** 1 Hubei Biopesticide Engineering Research Centre, Wuhan 430070, China; 88251420@webmail.hzau.edu.cn (H.L.); zhaoyuan.wu@nberc.com (Z.W.); 2 State Key Laboratory of Agricultural Microbiology, College of Veterinary Medicine, Huazhong Agricultural University, Wuhan 430070, China; 2018302110164@webmail.hzau.edu.cn (C.W.); 2017302110131@webmail.hzau.edu.cn (W.L.); xiaodanLi@webmail.hzau.edu.cn (X.L.); 97wgy@webmail.hzau.edu.cn (G.W.); dongwq@webmail.hzau.edu.cn (W.D.); 3 Key Laboratory of Preventive Veterinary Medicine in Hubei Province, The Cooperative Innovation Center for Sustainable Pig Production, Wuhan 430070, China; 4 Key Laboratory of Development of Veterinary Diagnostic Products, Ministry of Agriculture of the People’s Republic of China, Wuhan 430070, China; 5 International Research Center for Animal Disease, Ministry of Science and Technology of the People’s Republic of China, Wuhan 430070, China

**Keywords:** apigenin, *Streptococcus suis*, ampicillin, STSLS, inflammation

## Abstract

As an important zoonotic pathogen, *Streptococcus suis* (*S. suis*) can cause a variety of diseases both in human and animals, especially Streptococcal toxic shock-like syndrome (STSLS), which commonly appears in severe *S. suis* infection. STSLS is often accompanied by excessive production of inflammatory cytokines, which is the main cause of host death. Therefore, it is urgent to find a new strategy to relieve the damage caused by STSLS. In this study, we found, for the first time, that apigenin, as a flavonoid compound, could combine with ampicillin to treat severe *S. suis* infection. Studies found that apigenin did not affect the growth of *S. suis* and the secretion of suilysin (SLY), but it could significantly inhibit the hemolytic activity of SLY by directly binding to SLY and destroying its secondary structure. In cell assays, apigenin was found to have no significant toxic effects on effective concentrations, and have a good protective effect on *S. suis*-infected cells. More importantly, compared with the survival rate of *S. suis*-infected mice treated with only ampicillin, the survival rate of apigenin combined with an ampicillin-treated group significantly increased to 80%. In conclusion, all results indicate that apigenin in combination with conventional antibiotics can be a potential strategy for treating severe *S. suis* infection.

## 1. Introduction

*S. suis* can cause many disorders, such as septicemia, meningitis, arthritis, and endocarditis, in both humans and pigs and has a high mortality rate. According to incomplete statistics, since the first case was found in 1968, more than 1600 cases of human infection of *S. suis* have been reported [1]. *S. suis* is an emerging infectious pathogen. The main reason for concern with this pathogen is that it has a high potential for zoonosis when in close contact with infected pigs or contaminated raw pork products or when eating undercooked pork products [2]. Among the known serotypes of *Streptococcus suis*, serotype 2 (SS2) is the most common serotype in pigs and humans, and it has also been reported worldwide [3]. *S. suis* is a serious pathogen that causes meningitis in Vietnam, Thailand, and Hong Kong [4,5]. The survivors of meningitis caused by *S. suis* often have severe sequelae such as deafness [6]. In 2005, 215 human cases of *S. suis* were reported in Sichuan Province. It has been reported that Streptococcal toxic shock-like syndrome (STSLS) caused by *S. suis* shows serious consequences, including acute high fever, hypotension, shock, blood spots, multiple organ dysfunction, and even death. *S. suis* infection has also caused serious public health problems. There is an urgent need to better understand the pathological factors associated with *S. suis* infection and take effective action to minimize the social burden caused by *S. suis* infection.

Previous studies have demonstrated that suilysin (SLY), which is an important virulence factor, plays a significant role in the pathogenesis of SS2 infection and induces an inflammatory response [7,8]. SLY was reported to lyse red blood cells to release hemoglobin along with *S. suis* cell wall components to increase the levels of proinflammatory mediators in vivo [9]. In some previous studies, the virulence of SS2 was reduced by flavonoids, including apigenin and fisetin, which can weaken the pathogenicity of SS2 by inhibiting the hemolytic activity of SLY [10,11,12]. *S. suis* with high levels of SLY is more likely to cause mortality in infected models when compared with nonvirulent strains, indicating that the pathogenicity of *S. suis* can be enhanced by increasing the production of SLY [13,14]. In addition, NLRP3 inflammasome activation induced by SS2 strain SC19 with increased SLY production is the main reason for the excessive inflammatory response and multi-organ damage by STSLS [15]. Although the role of SLY and its contribution to human SS2 infection is not fully understood, it has been reported that SLY-positive strains can cause more severe symptoms than SLY-negative strains [16,17]. Furthermore, at the transcriptional level, upregulation of sly gene expression can improve the infection ability of SS2 [16,18]. Therefore, finding a novel anti-virulence compound that can suppress the activity of sly will greatly relieve inflammation caused by *S. suis* with high levels of SLY.

STSLS is characterized by a bacterial burden, an inflammatory cytokine storm, multiple system organ failure, and acute death of the final host [19]. Clinical retrospective studies have shown that the levels of interleukin (IL)-1β, IL-6, IL-8, IL-12, tumor necrosis factor-α (TNF-α), and interferon-γ (IFN-γ) in the blood of patients with STSLS were much higher than those in the blood of people without STSLS [19]. Further studies have shown that it is necessary to induce an excessive inflammatory response in STSLS [20,21]. It is worth noting that the degree of the excessive inflammatory response and organ damage caused by SC19 is much higher than that of the classical virulent strain P1/7, which may also be the main cause of the high mortality rate. Therefore, inhibiting the inflammatory cytokine storm caused by *S. suis* is the key to curing streptococcosis.

Several reports have shown that the targeted toxicity of traditional Chinese medicine can show therapeutic potential [22,23]. Apigenin, as a flavonoid, can be isolated from celery. Some studies have shown that apigenin has anti-cancer, antioxidant, and anti-inflammatory effects [24,25,26]. We demonstrated that apigenin can bind to *S. suis* as the protein hemolysin (SLY) actively centers to inhibit their function. In particular, we found that combined treatment with apigenin at a later stage of infection greatly increased the survival rate of mice when compared with ampicillin treatment alone. These findings suggest that apigenin may be a good candidate for the treatment of *S. suis* infection.

## 2. Results

### 2.1. Apigenin Inhibits the Hemolytic Activity of SLY without Interfering with SS2 Growth

**Figure 1:**
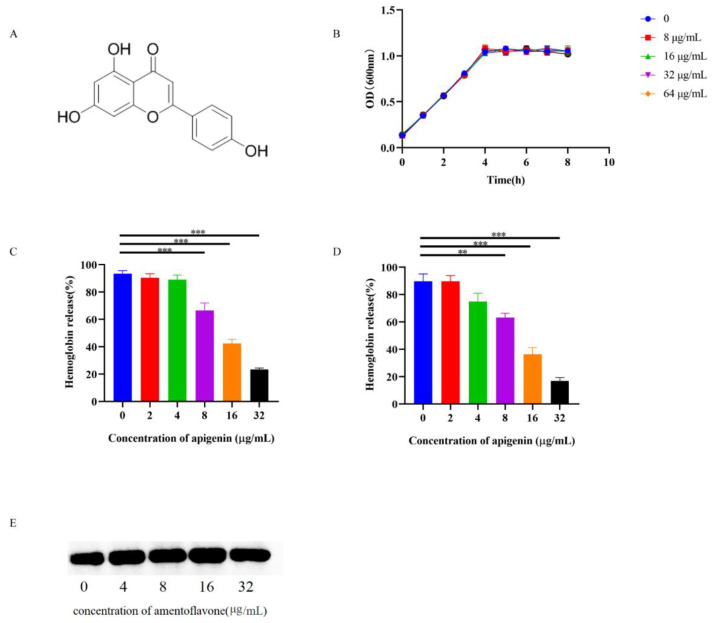
Apigenin inhibits the hemolytic activity of suilysin (SLY). (**A**) 2D structure of apigenin. (**B**) The growth curve of SC19 was determined. SC19 was cultured with 5% fetal bovine serum and treated with different concentrations of apigenin. (**C**) Hemolytic activity of supernatants from SC19 and apigenin co-culture system. (**D**) Effect of apigenin on hemolytic activity of purified SLY (100 ng/mL). (**E**) The expression of SLY was detected in the culture supernatant with or without apigenin. The data was obtained through three independent experiments. ** *p* < 0.01, *** *p* < 0.001 versus SS2 alone.

A growth curve was constructed to confirm that the growth of SS2 was not significantly inhibited by apigenin when the concentration of apigenin increased from 8 to 128 μg/mL (Figure 1B). As shown in Figure 1C, the supernatant of SC19 medium showed hemolytic activity as previously reported [22]. The hemolytic activity of the supernatant from the coculture system of SC19 and apigenin (8 to 32 μg/mL) was significantly lower than that of the SC19 culture alone, which showed that apigenin decreased the hemolytic activity of the SC19 culture supernatant in a dose-dependent manner. Therefore, the decreased hemolytic activity induced by apigenin may be due to the decreased expression of SLY or the decreased pore-forming activity of SLY in the culture supernatant. The culture supernatant was analyzed by using a Western blot. Notably, with increasing apigenin concentration (0 to 32 μg/mL), the expression of SLY in the culture supernatant did not change significantly (Figure 1D), indicating that apigenin reduced the hemolytic activity of SLY but not by inhibiting the expression of SLY. In order to better determine the neutralizing activity of apigenin, purified SLY was incubated with apigenin to verify that apigenin alone could inhibit the hemolytic activity of the SLY protein. The hemolytic activity of purified SLY decreased significantly with increasing apigenin concentration (8 to 32 µg/mL) (Figure 1E), indicating that apigenin directly inhibited the hemolytic activity of SLY.

### 2.2. Apigenin Safety Evaluation

**Figure 2:**
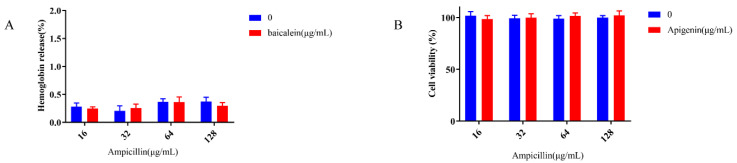
Effect of apigenin on the safety of ampicillin. (**A**) Hemolytic activity of ampicillin to the red blood cells (RBCs) in the absence or presence of apigenin. (**B**) Addition of apigenin exerts a negligible effect on the cytotoxicity of ampicillin in verocells.

In the clinic, the key problem with combination therapy is whether the toxicity of antibiotics increases when combined with adjuvants. Therefore, the hemolysis and cytotoxicity of ampicillin to mammalian cells in the presence and absence of apigenin were measured. The effects of a high level of apigenin (32 μg/mL) on the hemolysis of erythrocytes (RBCs) and the cytotoxicity of vero cells was negligible (Figure 2A,B).

### 2.3. Cytokines

**Figure 3:**
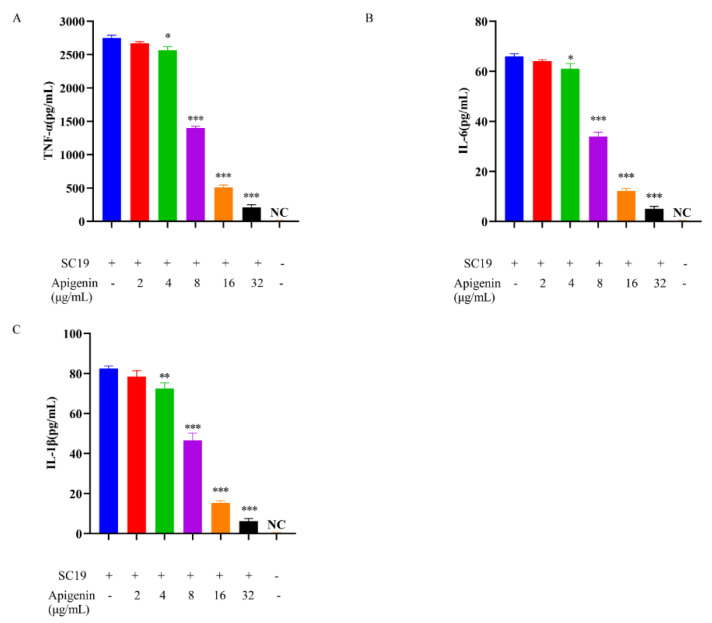
Apigenin reduced SS2-mediated cytokine production at the cellular level. (**A**) TNF-α, (**B**) IL-6, (**C**) IL-1β. Cells were incubated with SS2 (MOI  =  10:1) and different concentrations of apigenin for 6 h. ELISAs was used to determine the concentrations of TNF-α, IL-1 β, and IL-6. “NC” NC “represents no treatment with SC19. * *p*  <  0.05; ** *p*  <  0.01; *** *p*  <  0.001 versus SS2 alone.

*S. suis* infection can causes the host to produce several proinflammatory cytokines. It has been reported that STSLS was often accompanied with excessive production of inflammatory cytokines, including TNF-α, IL-6, and IL-1β. To evaluate the immunomodulatory activity of apigenin on *S. suis* infected macrophages, J774 cells were incubated with SS2 (multiplicity of infection [MOI]  =  10:1) and various concentrations of apigenin for 6 h. The level of TNF-α, IL-6, and IL-1β were measured by enzyme-linked immunosorbent assays (ELISAs). The levels of TNF-α (Figure 3A), IL-1β IL-6 (Figure 3B,C) in supernatants were significantly lower in SS2-infected cells treated with apigenin (2 to 32 μg/mL) than in untreated SS2-infected ones.

### 2.4. Identification of the Binding Sites between Apigenin and SLY

**Figure 4:**
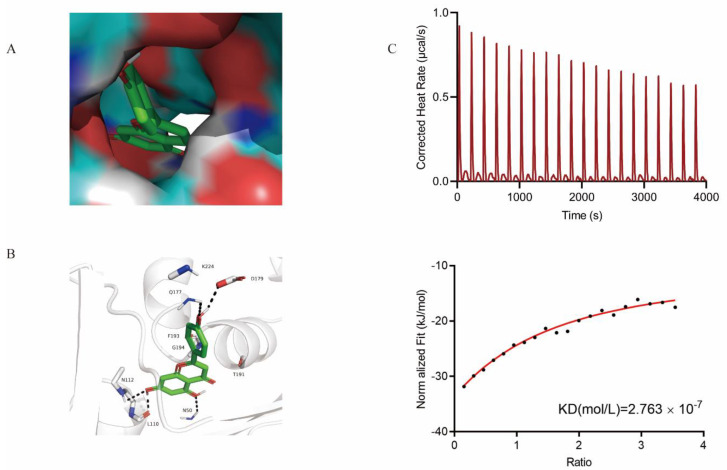
Based on the directly binding to the suilysin (SLY), apigenin could destroy hemolytic activity. (**A**) Apigenin was docked into the binding site of the SLY (Total view). (**B**) Apigenin and SLY binding site. (Detailed view). The apigenin was represented with green sticks. The hydrogen bond was shown in a black dotted line. (**C**) Isothermal titration calorimetry (ITC) was used to analyze the interaction between SLY and apigenin. 0.2 mmol/L of apigenin was dropped into 0.02 mmol/L of SLY in PBS buffer at 25 °C. Thermodynamic parameters were calculated, including the equilibrium dissociation constant (KD  =  2.763 × 10^−7^ mol/ L).

**Figure 5:**
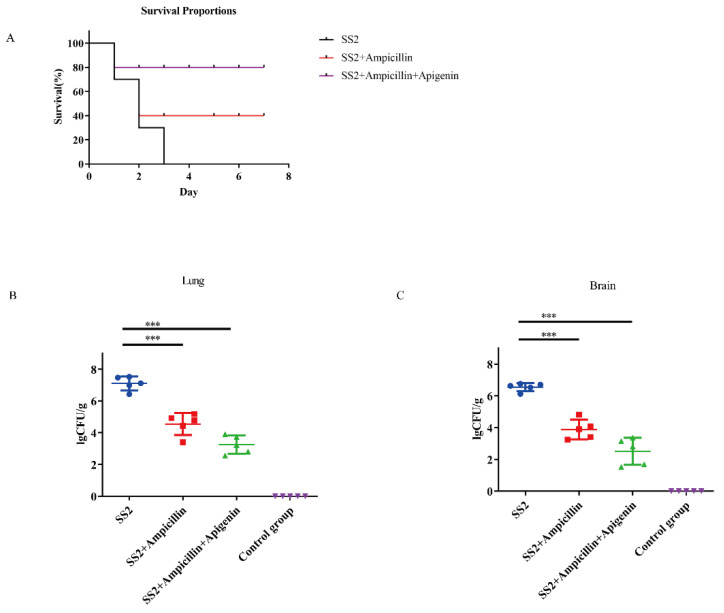
(**A**) The survival rates of apigenin + ampicillin and ampicillin cured a severely infected mice model. Bacterial burdens in the livers and spleens of the infected mice. Mice were intraperitoneally inoculated with 5 × 10^8^ CFU/mL of SC19. Bacterium number in the lung (**B**) and spleen (**C**) was counted at 8 h post-infection (two-tailed, unpaired *t*-tests, n = 5). *** *p*  <  0.001.

To find the appropriate binding site of apigenin to SLY, we used a molecular docking method, and the estimated binding energy was −5.77 kcal/mol. The hypothetical binding mode of apigenin in the binding site of SLY has been illustrated. In this model, we found that apigenin adopted a compact conformation to bind to the binding site of SLY (Figure 4A). Apigenin and the protein amino acid residues THR191, PHE193, GLY194, and LYS224 form strong hydrophobic interactions. Apigenin forms hydrogen bonding interactions with amino acid residues ASN50, LEU110, ASN112, GLN177, and ASP179, and these interactions make the protein-apigenin complex stable (Figure 5B). All of these interactions helped apigenin anchor to the binding site of SLY. Isothermal titration calorimetry (ITC) was used to determine the interaction between apigenin and SLY. This assay showed that the equilibrium dissociation constant (KD) between apigenin and SLY was 2.763 × 10^−7^ mol/L (Figure 3C), which implied that apigenin had a high affinity for SLY.

### 2.5. Apigenin Changed the Secondary Structure of SLY

Circular dichroism (CD) spectroscopy is an excellent method to detect the structural changes of proteins under different conditions [27]. In this study, we used CD spectroscopy to evaluate the effects of different concentrations of apigenin on the conformation of SLY. The calculated secondary structure is displayed in Table 1. Apigenin caused a change in the conformation of SLY. After the protein and apigenin were incubated together, the percentage of SLY in the α-helical conformation decreased, while the proportion of β-sheets and β-turns increased. These results indicated that apigenin caused conformational changes in SLY after binding to the protein.

### 2.6. Therapeutic Effects of Apigenin Combined with Ampicillin in SS2 SC19-Infected Mice

**Figure 6:**
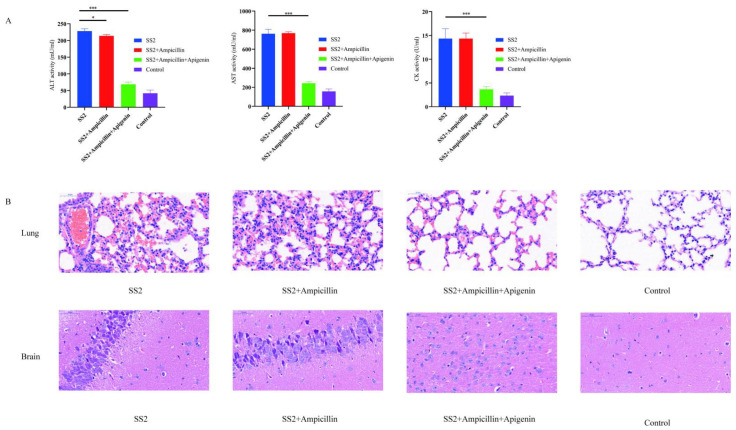
Tissue pathological changes of SC19-infected mice. The dose and interval of each treatment were 5 mg/kg and 12 h. (**A**) Blood levels of alanine transaminase (ALT), aspartate transaminase (AST), and creatine kinase (CK) at 6 h post-infection (two-tailed, unpaired *t*-tests, n = 5). (**B**) Pathological changes of lung and brain tissue after apigenin and ampicillin treatment. Apigenin alleviated tissue damage of infected mice. * *p*  <  0.05; *** *p*  <  0.001.

To determine the therapeutic effects of apigenin in vivo, a mouse model of severe *S. suis* SC19 infection was established. First, the protective effects of apigenin on the infected mice were evaluated by the survival rate. As shown in Figure 5A, the survival rate of apigenin combined with ampicillin-treated mice increased to 80% when compared with that of untreated infected mice, and the survival rate observed in the group of mice treated with only ampicillin was only 30%. We found that either ampicillin alone or in combination with apigenin significantly reduced the bacterial burden in the tissue (Figure 5B). Apigenin combined with ampicillin significantly relieved inflammation and pathological damage, including infiltration of inflammatory cells, alveolar interstitial congestion, and edema in the lungs and brains of the infected mice (Figure 6A). Moreover, we assessed the effects of apigenin and ampicillin on the levels of alanine transaminase (ALT), aspartate transaminase (AST), and creatine kinase (CK). Our results indicated that apigenin combined with ampicillin notably decreased the levels of blood biochemistry indicators in infected mice (Figure 6B). Therefore, these data indicate that the anti-inflammatory effects of apigenin are essential for improving the survival of severely infected mice.

## 3. Discussion

It has been reported that there are cytotoxins in different kinds of gram-positive bacteria. Cytotoxins, such as the drug lysin expressed by Streptococcus intermedius and aerolysin o expressed by *Clostridium perfringens* and *Listeria monocytogenes*, are necessary for bacteria to successfully infect the host [28,29,30,31]. The mechanism of conventional antibiotics is to destroy the basic functions of bacteria, such as cell wall synthesis, DNA replication, or protein synthesis [32]. However, with the increase in antibiotic resistance of many clinically relevant bacteria, it is necessary to urgently develop new classes of antibacterial drugs that are not affected by resistance mechanisms [33,34]. The development of drugs targeting virulence factors has become an important alternative for the treatment of infections caused by drug-resistant bacteria [35]. As an essential virulence factor, SLY was shown to activate high levels of the inflammasome, which plays an important role in STSLS [36]. In addition, SLY has also been identified to play an important role in meningitis caused by *S. suis* [14]. Therefore, targeting SLY, which is related to inflammation, is a novel strategy for the treatment of *S. suis* while relieving the development of antibiotic resistance in *S. suis*. Antiviral factors are superior to traditional antibiotics in two key aspects. First, they suppress the target genes necessary for basic metabolism. These genes usually play an important role in the pathogenic process and can allow bacteria to proliferate in the host. Second, drugs targeting virulence factors can specifically protect bacteria in the normal flora [35]. In this study, we examined alternatives to natural products to prevent and treat *S. suis* infections and, for the first time, combined an antitoxin and a clinical first-line antimicrobial to treat a severe bacterial infection.

Previous studies have suggested that apigenin can inhibit the production of α-hemolysin and alleviate the symptoms of *Staphylococcus aureus pneumonia* [37,38]. Unlike *S. aureus*, in this study, we found that apigenin significantly reduced the hemolytic activity of the SC19 culture supernatants and purified recombinant SLY but did not affect hemolysin expression. Moreover, apigenin had no effect on the growth of *S. suis* SC19 at a concentration that effectively inhibited the hemolytic activity of SLY, which indicates that apigenin had less selective pressure for survival than conventional antibacterial agents in the treatment of SS2 infection. CD spectra were used to analyze the secondary structure of SLY after apigenin treatment. It has been reported that a change in the secondary structure is related to the interaction between the protein and other components [39]. In addition, the results of molecular docking suggested that one potential binding site existed in the SLY protein that interacted with apigenin via hydrophobic interactions and hydrogen bonds. Finally, the Kd values from the Isothermal titration calorimetry (ITC) assay showed that apigenin could interrupt protein-receptor interactions by direct strong binding to SLY in vitro, which was the main reason for the anti-hemolysin activity of apigenin. We found that apigenin can directly target SLY to exert its antimicrobial effects and will not exert selective pressure on SS2 for survival. Therefore, apigenin is expected to be an effective candidate for the treatment of infections caused by *S. suis*. In order to prove this point, verifications were subsequently carried out at the animal and cellular levels. Consistent with the above expectation, apigenin treatment significantly alleviated cell damage after SS2 infection at a concentration that did not affect the growth of SS2. Although traditional antibiotics can play an effective role in eliminating *S. suis*, these antibiotics cannot improve the survival rate of infected mice by inhibiting excessive proinflammatory responses [40]. We found that the use of the first-line drug ampicillin in cases of nonresistant *S. suis* in a mouse model of severe infection maintained low rates of protection (40%). However, after the addition of apigenin, there was a clear improvement in survival (80%). Our results show that apigenin neutralizes hemolysin toxicity and reduces the inflammatory response, which is the primary cause of this phenomenon. This result suggests that, for some severely infected patients, apigenin in combination with first-line drugs may be a good choice.

Inflammation plays a significant role in protecting the human body from infection by various pathogens. However, excessive inflammation is not good for the body and may cause serious diseases [36]. Excessive inflammation could cause organ damage and accelerate disease progression, which is one of the serious consequences of *S. suis* infection. SLY was reported to play a significant role in inducing an excessive inflammatory response [15]. The severity of SS2 infection has a strong relationship with the host’s natural immune response. The host immune system can produce a large number of proinflammatory cytokines after stimulation by SS2, including TNF-α, IFN-γ, IL-1β, IL-6, and monocyte chemoattractant protein 1 (MCP-1) [19,41]. In addition, excessive inflammation plays a role in some clinical symptoms of SS2 infection, including meningitis, sepsis, septic shock, and sudden death [42]. Therefore, alleviating excessive inflammation is an important method to improve the consequences of SS2 infection. We explored the effects of apigenin on the anti-inflammatory activity of SS2 infection. The results showed that apigenin significantly inhibited the production of TNF-α, IL-1β, and IL-6 in the supernatant of J774 cells infected with SS2 in a dose-dependent manner. In addition, apigenin in combination with the first-line drug ampicillin decreased the levels of blood biochemical markers (ALT, AST, CK) and the bacterial load in the tissues of the mice infected with SS2 strain SC19, which contributed to the higher survival rate of infected mice when compared with the infected mice treated with the single anti-hemolysin compound fisetin in our study [11]. More importantly, the values of transaminases in infected mice were indicative of improvements in liver levels, which also suggested that apigenin may have an important hepato-reparative or hepato-protective effect. Moreover, it was reported that apigenin exhibited no hematological toxicity and had quite a limited toxic side effect [43]. We found that apigenin in combination with ampicillin did not increase blood or cell toxicity. In summary, our findings indicate that apigenin can provide a novel therapeutic approach to *S. suis* infection due to its anti-hemolysin activity. This study lays a foundation for developing apigenin into a new drug against *S. suis*.

## 4. Materials and Methods 

### 4.1. Bacterial Strains, Growth Conditions, and Apigenin Preparation

SS2 strain SC19 was isolated from the brains of dead pigs during an outbreak of *S. suis* in Sichuan Province in 2005 [44]. These *S. suis* strains were cultured in Todd-Hewitt broth (THB) or plated on tryptic soy agar (THA) (Summus Ltd., Shanghai, China) with 5% (*v/v*) fetal bovine serum (Sijiqing Ltd., Shanghai, China) at 37 °C. Apigenin was obtained from Topscience, and dimethyl sulfoxide (DMSO, Sigma-Aldrich, St. Louis, MO, USA) was used to dissolve the drugs.

### 4.2. Apigenin Assay on the Growth of SC19

The SC19 overnight culture containing 10% newborn bovine serum in THB was diluted into 10 mL of equal parts to a final concentration of 5 × 10^5^ CFU/mL, and a final concentration of apigenin (0, 8, 16, 32, 64, and 128 µg/mL) was added to the cell culture plate. In order to measure the effects of apigenin on SC19, an automatic microbial growth curve analysis system (Bioscreen C) was used to detect the growth of bacteria at an optical density of 600 nm (OD600) every 30 min [45].

### 4.3. Activity of Apigenin against the Hemolytic Activity of SLY

*S. suis* strain SC19 was cultured for 12 h at 37 °C. The cultures were centrifuged at 12,000 rpm for 15 min at 4 °C. Subsequently, the culture supernatant was collected and incubated for 30 min with final concentrations of apigenin at 0, 2, 4, 8, 16, and 32 µg/mL at 37 °C. Then, 2% defibrated sheep red blood was added and incubated at 37 °C for 30 min. Finally, the mixture was centrifuged at 1000 rpm for 5 min at 4 °C, and 200 µL aliquots of the supernatant were collected and measured with a BioSpectrometer (Eppendorf) at an optical density of 543 nm. The sample was treated with 2.5% Triton X-100 as a 100% cleavage control. The activity of apigenin against the hemolytic activity of SLY was evaluated by the ratio of the OD543 of each sample to that of the 100% cleavage control.

### 4.4. Preparation of the Recombinant SLY Protein and Anti-SLY Protein Hemolysis Assay

The prokaryotic expression plasmid pET-28a(+)-SLY was built by subcloning SLY cDNA into the pET-28a(+) vector (Novagen, Madison, WI, USA) using BamHI and NdeI restriction enzyme cutting sites. *E. coli* BL21 transformed with the recombinant plasmid was cultured in medium containing 0.2 mM isopropyl-β-d-thiogalactopyranoside (IPTG) and induced for 16 h at 16 °C. The resultant rSLY was purified by loading the supernatant of the bacterial cell lysates onto a His_6_-Ni-nitrilotriaceate (Ni-NTA) column. The antihemolytic activity of apigenin was directly evaluated by co-incubation with the purified protein (100 ng/mL) and apigenin at different concentrations (0, 2, 4, 8, 16, and 32 µg/mL), as described above.

### 4.5. Western Blotting Assay

The bacterial culture supernatant with or without apigenin was centrifuged at 12,000 rpm for 5 min at 4 °C. Mini protein tetra cells (Bio-Rad Laboratories, Inc., Hercules, CA, USA) were used for SDS-PAGE under reducing conditions. The sample was separated on a 12% separation gel at 120 V and then transferred to a polyvinylidene fluoride (PVDF) membrane. After sealing with 5% skimmed milk powder, the membrane was incubated with rabbit anti-sly primary antibody at a dilution of 1:1000 for 4 h. Then, the membrane was conjugated with horseradish peroxidase at a dilution of 10,000. Amersham ECL Western blotting detection reagents (GE Healthcare, Buckinghamshire, UK) were used to detect the protein bands on the membrane.

### 4.6. Safety Assessment

Two percent defibrillated sheep red blood cells were incubated with ampicillin (16–128 μg/mL) or apigenin (32 μg/mL) at 37 °C for 1 h. Phosphate-buffered saline (PBS, pH = 7.4) was used as a positive control and a negative control when in the presence or absence of 2.5% Triton X-100, respectively. A Fluostar Omega was used to measure the absorption of the released hemoglobin at 543 nm. The following formula was used to evaluate the hemolysis rate. Hemolysis (%) = [(OD576 sample − OD576 blank)/(OD576 2.5% Triton X-100 − OD576 blank)] × 100%. Cytotoxicity in VERO cells were performed by CCK8 assay (US Everbright^®^ Inc., Suzhou, China) by measuring the absorbance at 450 nm. Ampicillin (16–128 µg/mL) with apigenin (32 µg/mL) and 1 × 10^4^ cells were added to 96-well plates at the same time and cultured in DMEM with 10% heat inactivated FBS at 37 °C for 24 h. Then, CCK8 (US Everbright^®^ Inc., Suzhou, China) was added.

### 4.7. Enzyme-Linked Immunosorbent Assays (ELISAs) 

J774 cells were seeded in 6-well plates at a density of 1 × 10^6^ cells/well and incubated for 12 h. The cells were infected with SC19 with or without apigenin for 6 h. According to the kit manufacturer’s instructions, the levels of TNF-α, IL-1β, and IL-6 in the culture supernatant were determined by ELISAs kits (BioLegend, San Diego, CA, USA).

### 4.8. Homology Modeling and Molecular Docking

The amino acid sequence of *S. suis* SLY was searched by using the national ceter for biotechnology information search database (NCBI) protein database (http://www.ncbi.nlm.nih.gov/protein/ (accessed on 23 March 2021)) and the protein sequence was NC_012924.1. AutoDock Vina 1.5.6 was used to conduct the molecular docking study of SLY and apigenin, which improved the docking speed and accuracy through the new scoring function. ChemBioDraw Ultra 14.0 and ChemBio3D Ultra 14.0 were used to draw the 2D and 3D structures of apigenin. The docking input file was obtained through AutoDock tools 1.5.6 [46,47].

### 4.9. Isothermal Titration Calorimetry (ITC) Assay

The interaction of the SLY protein and apigenin was determined by calorimetry using affinity ITC (TA NANO ITC) in vitro. The purified SLY protein (0.02 mmol/L) and apigenin (0.2 mmol/L) were dissolved in phosphate buffer saline (PBS) (pH 7.4). apigenin was injected into the sample cell filled with the purified SLY protein, and the injection was repeated 20 times with an equilibrium interval of 200 s. The experiment was conducted at 25 °C. The equilibrium dissociation constant (KD) was determined by nanoAnalyzer software.

### 4.10. Circular Dichroism Analysis

At 37 °C, apigenin was co-incubated with purified sly (0.5 μg/mL) for 1 h. A circular dichroism (CD) spectrophotometer (MOS-500, Bio-Logic, France) was used to determine the secondary structure of SLY at room temperature (25 °C). The scanning wavelengths and rate were 190 to 250 nm and 50 nm/min, respectively, and the bandwidth was 1.0 nm. The Bestsel web server was used to analyze the secondary structure of SLY [48].

### 4.11. Establishment of the S. suis 2 SC19-Infected Mouse Model In Vivo

Seven-week-old female BALB/c mice were purchased from China Three Gorges University to establish a mouse model of SS2 SC19 serious infection. Animal experiments conformed to animal ethical procedures, and all experiments were conducted under the guidance of the Protection, Supervision, and Control Committee of Animal Experiments of Huazhong Agricultural University (HZAUMO-2021-0008). SS2 SC19 was transferred into TSB medium at a dilution of 1:100 and cultured at 37 °C until the OD600 = 0.6. The bacteria were collected after centrifugation at 10,000 rpm for 10 min at 4 °C and then suspended in PBS (pH 7.4). In the survival rate assay, the concentration of SC19 in intraperitoneally infected mice was 1.25 × 10^9^ cells/mL (200 µL). After four hours of infection, the mice were treated with apigenin (5 mg/kg) and ampicillin (5 mg/kg) or ampicillin (5 mg/kg) alone by intraperitoneal injection. The interval of each treatment was 12 h. The control group (10 per group) was injected with an equal volume of phosphate buffer saline (PBS) (pH 7.4). Based on assay data, the survival curve of the mice was constructed.

Additionally, mice (5 per group) were intraperitoneally infected with 200 µL of SC19 at a concentration of 2.5 × 10^8^ cells/mL, and then apigenin or ampicillin was injected as described above. The control group was injected with PBS (pH 7.4). At 12 h post injection, the cardiac blood of anesthetized mice was collected to analyze the effects of apigenin on the levels of blood biochemistry parameters (alanine transaminase ALT, aspartate transaminase AST, and creatine kinase CK) of the SC19-infected mice. The lung, spleen, kidney, and liver were ground, diluted, and inoculated onto a TSA plate containing 5% fetal bovine serum. The samples were cultured overnight at 37 °C, and then the bacteria were counted. Finally, the lung and brain tissues were immobilized in 4% paraformaldehyde to analyze pathological changes.

### 4.12. Statistical Analysis

All experimental data (n ≥ 3) are expressed as the means ± SD. GraphPad Prism 9.0.2 (GraphPad Software, San Diego, CA, USA) was used for statistical analysis using a two-tailed unpaired t-test.

## 5. Conclusions

In the present study, we found that apigenin could relieve *S. suis* infections by targeting SLY and inhibiting inflammation. In addition, it was found that the combination of apigenin and ampicillin had a good therapeutic effect in the assay to combine anti-virulence drugs and clinical antibacterial drugs to treat severe *S. suis* infections. The results indicated that apigenin may be a promising therapeutic candidate for *S. suis* infection. Meanwhile, the phenomenon suggested that it was particularly important to strengthen the monitoring of an inflammatory response while antibacterial treatment for some bacterial infections could cause a severe inflammatory response.

## Figures and Tables

**Table 1:** Determination of secondary structure components of suilysin (SLY) treated with apigenin at different concentrations.

Concentration of Apigenin (μg/mL)	Content (%)				NRMSDa
	α-Helix	β-Sheet	β-Turns	Others	
0 (control)	12.1	41.4	11.5	35	0.06575
32	0	45.6	19.1	35.3	0.15325

NRMSDa, normalized root-mean-square deviation.

## Data Availability

The data presented in this study are available on request from the corresponding author.

## Sample Availability

Samples of the compounds are available from the authors.
